# Expansions of chemosensory gene orthologs among selected tsetse fly species and their expressions in *Glossina morsitans morsitans* tsetse fly

**DOI:** 10.1371/journal.pntd.0008341

**Published:** 2020-06-26

**Authors:** Joy M. Kabaka, Benson M. Wachira, Clarence M. Mang’era, Martin K. Rono, Ahmed Hassanali, Sylvance O. Okoth, Vincent O. Oduol, Rosaline W. Macharia, Grace A. Murilla, Paul O. Mireji

**Affiliations:** 1 Biotechnology Research Institute—Kenya Agricultural and Livestock Research Organization, Kikuyu, Kenya; 2 Department of Biochemistry, Microbiology and Biotechnology, School of Pure and Applied Sciences, Kenyatta University, Ruiru Campus, Nairobi, Kenya; 3 Department of Chemistry, School of Pure and Applied Sciences, Kenyatta University, Ruiru Campus, Nairobi, Kenya; 4 Department of Biochemistry and Molecular Biology, Egerton University, Njoro Campus, Egerton, Kenya; 5 Centre for Geographic Medicine Research—Coast, Kenya Medical Research Institute, Kilifi, Kenya; 6 Department of Biochemistry, University of Nairobi, Nairobi, Kenya; 7 Center for Bioinformatics and Biotechnology, University of Nairobi, Nairobi, Kenya; Seattle Biomedical Research Institute, UNITED STATES

## Abstract

Tsetse fly exhibit species-specific olfactory uniqueness potentially underpinned by differences in their chemosensory protein repertoire. We assessed 1) expansions of chemosensory protein orthologs in *Glossina morsitans morsitans*, *Glossina pallidipes*, *Glossina austeni*, *Glossina palpalis gambiensis*, *Glossina fuscipes fuscipes* and *Glossina brevipalpis* tsetse fly species using Café analysis (to identify species-specific expansions) and 2) differential expressions of the orthologs and associated proteins in male *G*. *m*. *morsitans* antennae and head tissues using RNA-Seq approaches (to establish associated functional molecular pathways). We established accelerated and significant (P<0.05, λ = 2.60452e-7) expansions of gene families in *G*. *m*. *morsitans* Odorant receptor (Or)71a, Or46a, Ir75a,d, Ionotropic receptor (Ir) 31a, Ir84a, Ir64a and Odorant binding protein (Obp) 83a-b), *G*. *pallidipes* Or67a,c, Or49a, Or92a, Or85b-c,f and Obp73a, *G*. *f*. *fuscipes* Ir21a, Gustatory receptor (Gr) 21a and Gr63a), *G*. *p*. *gambiensis* clumsy, Ir25a and Ir8a, and *G*. *brevipalpis* Ir68a and missing orthologs in each tsetse fly species. Most abundantly expressed transcripts in male *G*. *m*. *morsitans* included specific Or (Orco, Or56a, 65a-c, Or47b, Or67b, GMOY012254, GMOY009475, and GMOY006265), Gr (Gr21a, Gr63a, GMOY013297 and GMOY013298), Ir (Ir8a, Ir25a and Ir41a) and Obp (Obp19a, lush, Obp28a, Obp83a-b Obp44a, GMOY012275 and GMOY013254) orthologs. Most enriched biological processes in the head were associated with vision, muscle activity and neuropeptide regulations, amino acid/nucleotide metabolism and circulatory system processes. Antennal enrichments (>90% of chemosensory transcripts) included cilium-associated mechanoreceptors, chemo-sensation, neuronal controlled growth/differentiation and regeneration/responses to stress. The expanded and tsetse fly species specific orthologs includes those associated with known tsetse fly responsive ligands (4-methyl phenol, 4-propyl phenol, acetic acid, butanol and carbon dioxide) and potential tsetse fly species-specific responsive ligands (2-oxopentanoic acid, phenylacetaldehyde, hydroxycinnamic acid, 2-heptanone, caffeine, geosmin, DEET and (cVA) pheromone). Some of the orthologs can potentially modulate several tsetse fly species-specific behavioral (male-male courtship, hunger/host seeking, cool avoidance, hygrosensory and feeding) phenotypes. The putative tsetse fly specific chemosensory gene orthologs and their respective ligands provide candidate gene targets and kairomones for respective downstream functional genomic and field evaluations that can effectively expand toolbox of species-specific tsetse fly attractants, repellents and other tsetse fly behavioral modulators.

## Author summary

Tsetse flies are insect vectors of sleeping sickness in humans and nagana in livestock in sub-Sahara Africa. Tsetse flies identify their hosts (preferred and non-preferred) by detecting and processing odor cues emitted by the hosts in their environment. Tsetse flies use chemosensory proteins and associated pathways in their antennae to identify these cues. In this study, we identified expansions of these chemosensory protein in six tsetse fly species (*Glossina morsitans morsitans*, *Glossina pallidipes*, *Glossina austeni*, *Glossina palpalis gambiensis*, *Glossina fuscipes fuscipes* and *Glossina brevipalpis)* with different known hosts. We also identified potential ligands to these proteins based on fruit fly (*Drosophila melanogaster*) orthologs. With *G*. *m*. *morsitans* as an example, we identified the proteins and associated molecular pathways preferentially expressed in tsetse fly antennae. These proteins may be responsible for the tsetse fly species-specific host discrimination, with the ligands eliciting species-specific behavioral responses in the flies. The expressed orthologs may be functionally important in odor detection in tsetse fly and lay down useful groundwork for downstream functional genomics R&D for more effective tsetse fly species-specific odor attractants and repellents for routine tsetse fly control operations.

## Introduction

Human African Trypanosomiasis (HAT) constitutes one of the most neglected tropical diseases (NTDs) with devastating health and economic consequences in sub-Sahara Africa [1,2]. On the other hand, African Animal Trypanosomiasis (AAT) is rampant in livestock inhabiting tsetse-infested areas throughout the continent. The AAT cause death of about three million cattle each year [3], and in terms of agricultural Gross Domestic Product (GDP), loss of about US$ 4.75 billion per year [3]. The HAT and AAT causative trypanosomes are transmitted by different groups of tsetse species. Tsetse control is considered an effective approach and constitutes the corner stone in trypanosomiasis suppression [4,5]. Tsetse fly species belong to *Glossina* genus and are generally restricted to sub-Saharan Africa. Twenty-three species and eight sub-species of tsetse flies are recognized [6,7]. These species are divided into Morsitans, Palpalis and Fusca clade sub-genera, described by respective savanna, riverine/lacustrine and forest ecological niches they occupy. The Morsitans group consists of five species that include *Glossina morsitans morsitans* and *Glossina pallidipes* restricted to savannah grassland and *Glossina austeni* occupying coastal woodlands [8]. This group is adapted to drier habitats than Palpalis and Fusca [9] and preferentially feeds on livestock and wildlife. They are thus important vectors of African Animal Trypanosomiasis (AAT) also known as nagana. On the other hand, Palpalis group consists of five species, including *Glossina palpalis gambiensis* and *Glossina fuscipes fuscipes* in West, Central and East Africa. These species are predominant vectors of Human African Trypanosomosis (HAT), also known as sleeping sickness, despite their preferential predilection to feeding on reptiles and ungulates. Fusca group consist of 13 species largely inhabiting damp evergreen forests of West Africa (except *Glossina brevipalpis*) and are mainly associated with livestock. *Glossina brevipalpis* is of limited medical and agricultural significance and occurs discontinuously in other parts of sub-Saharan Africa [6].

These tsetse fly species exhibit different olfactory uniqueness, which partly accounts for their gradation of preferences for their particular hosts. This olfactory uniqueness (and visual responses) has been exploited in designing effective tsetse fly bait technologies that consist of synthetic blends of attractants and repellents that mimic those of their natural hosts and non-hosts respectively [10–13]. These technologies are especially applicable for *G*. *m*. *morsitans* and *G*. *pallidipes* but not *G*. *austeni* (among savanna species) [14] and palpalis group. For example, *G*. *pallldipes*, *G*. *m*. *morsitans* and to some extent *G*. *brevipalpis* are attracted to traps baited with POCA (3-n-propylphenol, 1-octen-3-ol, 4-cresol and acetone) and to which *G*. *austeni* poorly responds [15–17]. Molecular bases of these natural differential responses are poorly understood but may be underpinned by differences in their chemosensory apparatus. The chemosensory apparatus facilitate reception of odorants and tastants, and consist of Odorant-binding proteins (Obps), Odorant-degrading enzymes (Odes), Odorant receptors (Ors), Ionotropic receptors (Irs), Gustatory receptors (Grs), Chemosensory proteins (Csps), Sensory neuron membrane proteins (Snmps) and CD36-like pheromone sensors [18–24]. These chemosensory proteins mediate decoding of ecological odors and odorant specific behavioral responses in insect hosts. These responses include seeking for hosts, location of oviposition sites, searching for mates, and detecting and escaping from potential predators. The Obp transport pheromone molecules and general odorants to Ors [25]. The Ors are odorant-gated ion channels composed of an odorant-binding subunit and olfactory co-receptor Orco [26,27]. The Irs have higher specificity to volatiles than Ors, detecting specific variety of odors, such as acids, aldehydes, amines and humidity [20,28]. The Ir25a and Ir8a are putative conserved Ir co-receptors [23]. The Grs discern odor tastes and contact pheromones [29]. Only two Snmp subfamilies (Snmp 1 and Snmp 2) have been identified in insects, where Snmp1 is expressed in pheromone-sensitive Olfactory Receptor Neurons (ORNs) while Snmp 2 is expressed in supporting cells [30–32]. Some of these chemosensory proteins are present in non-canonical chemosensory organs, such as legs [33,34], wings [35,36] and pheromone glands [37], where only a subset of Irs are specifically expressed in *D*. *melanogaster* antennae [20]. Among tsetse flies, genomes of *G*. *pallidipes*, *G*. *m*. *morsitans*, *G*. *austeni*, *G*. *p*. *gambiensis*, *G*. *f*. *fuscipes* and *G*. *brevipalpis* (representative of the different clades/sub-general) have been sequenced [38], and their respective chemosensory proteins annotated [39–41].

Here we report on 1) expansions of chemosensory protein orthologs in six tsetse fly species/subspecies (*G*. *pallidipes*, *G*. *m*. *morsitans*, *G*. *austeni*, *G*. *p*. *gambiensis*, *G*. *f*. *fuscipes* and *G*. *brevipalpis*) to identify species-specific expansions and 2) differential expressions of these and associated proteins in antennae and head tissues *G*. *m*. *morsitans* to establish probable functional pathways influencing host seeking behaviors in this specie.

## Materials and methods

### Differential expansions of *D*. *melanogaster* chemosensory gene orthologs among tsetse flies

We obtained complete *D*. *melanogaster* gene set release 79 (*Drosophila_melanogaster*. BDGP6.pep.all.fa) from Ensembl project [42] in fasta format. We then isolated *D*. *melanogaster* chemosensory genes from the gene set by searching and retrieving flybase [43] chemosensory gene IDs in the gene set using “Odorant receptor”, “Gustatory receptor”, “Ionotropic receptor”, “Odorant-binding protein”, “Sensory neuron membrane protein” and “Glutamate receptor” Linux bash regular expressions. For Csp orthologs, we extracted *D*. *melanogaster* IDs from Macharia et al., (2016) [40]. We separately obtained VectorBase Release VB-2019-02 homologs (gene trees) of disease vectors from VectorBase database [44] in OrthoXML formats. The gene trees were pre-computed by Gene Orthology/Paralogy prediction pipeline in VectorBase [44] that identified gene duplications within species and speciation events. We probed the VectorBase homologs for ortholog groups (gene families) with the *D*. *melanogaster* chemonsensory genes (flybase IDs) to identify their respective tsetse flies (*G*. *austeni*, *G*. *f*. *fuscipes*, *G*. *p*. *gambiensis*, *G*. *brevipalpis*, *G*. *pallidipes* and *G*. *m*. *morsitans*) orthologs. We identified presence of the individual genes in each gene family (ortholog group) and species. Gene families with accelerated gene expansions were pre-computed through Computational Analysis of gene Family Evolution (CAFE) [45] in VectorBase [44]. We considered the VectorBase [44] pre-computed gene expansions/contractions reliable since they are 1) community reviewed and adopted and with stable ortholog IDs and 2) regularly updated (with new gene-sets and genomes). We also conducted Principal Component Analysis (PCA) in R using FactoMineR and Factoextra packages with species-specific gene counts as input data to establish relationship between the expanded/contracted chemosensory genes (Ors, Irs, Grs and Obps) and tsetse species.

### Transcriptional expression of *D*. *melanogaster* chemosensory gene orthologs in male *G*. *m*. *morsitans*

We employed high throughput Illumina based RNA-Seq approach to establish expression profiles of the *D*. *melanogaster* chemosensory gene orthologs in male *G*. *m*. *morsitans*. We established expression levels of the orthologs in the antennae and in relation to the head libraries. We isolated and sequenced RNA from antennae or head tissues from colony reared *G*. *m*. *morsitans* as described previously [46]. Briefly, we fed teneral male *G*. *m*. *morsitans* (1–3 days old) on defibrinated bovine blood meal (their initial blood meal post-eclosion) (commercially supplied by Hemostat Laboratories, Dixon, CA, USA) to putatively prime their chemosensory system. We then extracted their antennae in two independent biological replicates (from 50 flies each) using liquid nitrogen-based method of Menuz et al. (2014) [47] 72 hrs post-feeding. We envisaged that the 72 hrs deprivation of blood meal (food) would biologically prime potential host seeking chemosensory apparatus in the flies and enhance RNA-seq detection of chemosensory gene expressions, specifically those associated with hunger/host seeking.

The *G*. *m*. *morsitans* show marked die1changes in their biting activity in the field, with their peak activity in the morning and afternoon [48,49]. We thus snap froze individual tsetse flies in liquid nitrogen in the morning (09:30 hrs) and carefully hand-dissected their antennae from the head into 1.5 ml microfuge tubes kept cold in liquid nitrogen. We then isolated RNA by mechanically crushing the antennae with disposable RNAseq-free plastic pestles in TRIzol reagent (Invitrogen, Carlsbad, USA) following the manufacturer’s protocol. We removed traces of potential carry over DNA (that could potentially confound our RNA-Seq analysis) by digesting possible contaminating genomic DNAs (gDNA) in the total RNA using TURBO DNase (Ambion life technologies, TX, USA) following manufacturer’s instructions. We confirmed removal of the gDNA from total RNA by qualitative assessment of PCR amplicons from final RNA samples using tsetse fly specific *beta*-*tubulin* gene primers as documented in Bateta et al. (2017) [46]. We verified quality and integrity of RNA samples using Agilent Bioanalyzer 2100 (Agilent, Palo Alto, CA, USA) following manufacturer’s instructions. cDNA was then generated from the RNA using Illumina TruSeq RNA Sample Preparation Kit *(*Illumina, Hayward, CA, USA) and the cDNA (75 bp single-end read) and sequenced on Illumina HiSeq 2500 at Yale University Center of Genome Analysis (YCGA), New Haven, CT, USA. We similarly prepared head transcriptomes from two independent biological replicates (50 flies each) from 72 hrs starved 40 days old males. We deposited all transcriptome sequences at the Sequence Read Archive (SRA) under study accession numbers PRJNA343267 and PRJNA343269 for the antennae and head libraries respectively.

### Expression profiles of *D*. *melanogaster* chemosensory gene orthologs in male *G*. *m*. *morsitans* antennae and head libraries

We established quality of the reads in each individual transcriptome library using FastQC (Babraham Bioinformatics) software package (http://www.bioinformatics.babraham.ac.uk/projects/fastqc/)). We then used the FastQC results to clean (trimm) the reads using CLC genomic workbench version 10 software (CLC Bio, Aarhus, Denmark) through settings that permitted 1) removal of low quality sequences (limit = 0.05), 2) removal of ambiguous nucleotides (maximum 2 nucleotides allowed), 3) removal of terminal nucleotides (10 nucleotides from the 5’ end and 1 nucleotide from the 3’ end) and 4) removal of sequences on length (minimum length 15 nucleotides, maximum length 1000 nucleotides). We then mapped the cleaned reads on to *G*. *m*. *morsitans* transcripts gene-set version 1.9 from Vectorbase [44] using CLC genomic workbench version 10 software (CLC Bio, Aarhus, Denmark) thorough settings that permitted 1) mismatch cost of 2, 2) insertion/deletion cost of 3, 3)length fraction of 0.8, 4) similarity fraction of 0.8, 5) maximum number of reads per hit of 10, and 6) strand specificity set as both strands.

From the mappings, we established reads mapping per transcript and reads per kilobase of transcripts per Million mapped reads (RPKM), a normalized index of relative gene expression associated with each transcript (including chemosensory genes) in the gene-set for individual transcriptomes [50]. We then established differentially expressed transcripts between the antennae and the head transcriptomes by comparing the reads mapped in the genes sets from respective transcriptomes using edgeR software [51,52]. We considered transcripts validly differentially expressed if they had at least two-fold changes, p-value corrected False Detection Rate (FDR) < 0.05 and one Counts Per Million (CPM) coverage to mitigate against type I statistical errors. We then determined antennae or head enriched molecular processes using canonical Gene Set Enrichment Analysis (GSEA) using WEB-based GEne SeT AnaLysis Toolkit (WebGestalt) [53]. Since WebGestalt database did not include tsetse flies, but *D*. *melanogaster* gene set, we obtained homologs of the entire *G*. *m*. *morsitans* gene-set in *D*. *melanogaster* through Basic Alignment Search Tool (BLAST) analysis of protein sequences (Blastp) [54] of the *G*. *m*. *morsitans* gene-set against those of *D*. *melanogaster* and accepted hits with e-value < 0.001 as significantly homologous. We then used these *D*. *melanogaster* homologs as proxy in WebGestalt to assess enrichment of their associated *G*. *m*. *morsitans* homologs. We used the FDR corrected p-value ranked *D*. *melanogaster* homolog gene-sets of differentially expressed *G*. *m*. *morsitans* transcripts as input for the analysis [55]. We considered selection of 5–2000 Entrez Gene IDs, FDR < 0.05, 1000 permutations and 20 categories with the outputted leading-edge genes default parameters for the analysis. Through GSEA, we separated and identified significantly enriched non-redundant biological processes, cellular components and molecular function Gene Ontology (GO) terms, Kyoto Encyclopedia of Genes and Genomes, KEGG, PANTHER, Reactome, pathways and Database of Protein, Chemical and Genetic Interactions (BioGRID) network [56–61]. Next, we identified antennae or head (tissue) specific chemosensory genes by mapping the global most differentially (based on fold change) and abundantly (based on CPM) or significantly expressed (based on p-value) transcripts in MA or volcano plots respectively using edgeR software package [52,62] in R software [63]. We considered chemosensory genes with fold changes (FC) ≥ 1.25 as of chemosensory biological significance as previously documented [64].

## Results

### Expansions of chemosensory gene orthologs among tsetse fly species

We identified 60 each of Ors, Irs or Grs, 51 Obps, seven GluR and two Snmps (excluding isoforms) in *D*. *melanogaster* [43] and four Csps [40], with 58, 34, 13, 22, 2 and 3 orthologs (VectorBase gene trees, Release VB-2019-02) [44] respectively among the tsetse fly species (S1 Table). Café gene expansion analysis [45] revealed significant (P<0.05, λ = 2.60452e-7) accelerated expansions of several gene families/clusters including VBGT00190000010263 (Or71a and Or46a), VBGT00190000009736 (Ir75a,d, Ir31a, Ir84a and Ir64a) and VBGT00190000009994 (Obp83a-b) in *G*. *m*. *morsitans*, VBGT00840000047907 (Or67a,c, Or49a, Or92a, Or85b-c,f) and VBGT00190000013627 (Obp73a) in *G*. *pallidipes*, VBGT00190000012412 (Ir21a) and VBGT00190000010879 (Gr21a and Gr63a) carbon dioxide receptors orthologs [65] in *G*. *f*. *fuscipes*, VBGT00820000046003 (clumsy, Ir25a and Ir8a) in *G*. *p*. *gambiensis* and VBGT00190000013104 (Ir68a) in *G*. *brevipalpis* (S1 Table). No gene families were significantly expanded in *G*. *austeni*. We also identified several orthologs that were missing/absent in specific tsetse fly species (S1 Table). The Ir76b ortholog was absent in four tsetse fly species (*G*. *p*. *gambiensis*, *G*. *m*. *morsitans*, *G*. *pallidipes* and *G*. *brevipalpis*) while Gr33a was missing in *G*. *brevipalpis*. Both Gr32a and Gr68a were missing in *G*. *brevipalpis* and *G*. *m*. *morsitans*. The Gr64a-f, Gr5a, Gr43a, Obp56a/d/e and Or71a orthologs were absent in all tsetse fly species. The Snmp1, Or67d and Obp19a and Orco ortholog appeared to be conserved across all tsetse fly species. Our PCA analysis revealed a general positive correlation between tsetse species across four chemosensory groups (Ors, Irs, Grs or Obps). Additionally, Gr and Ir orthologs appeared to be positively correlated (S1 Fig panels B2 and B3) in relation to a unique *G*. *m*. *morsitans* cluster (S1 Fig panels A2 and A3).

### Expression profiles of chemosensory ortholog transcripts in male *G*. *m*. *morsitans* antennae

**Fig 1:**
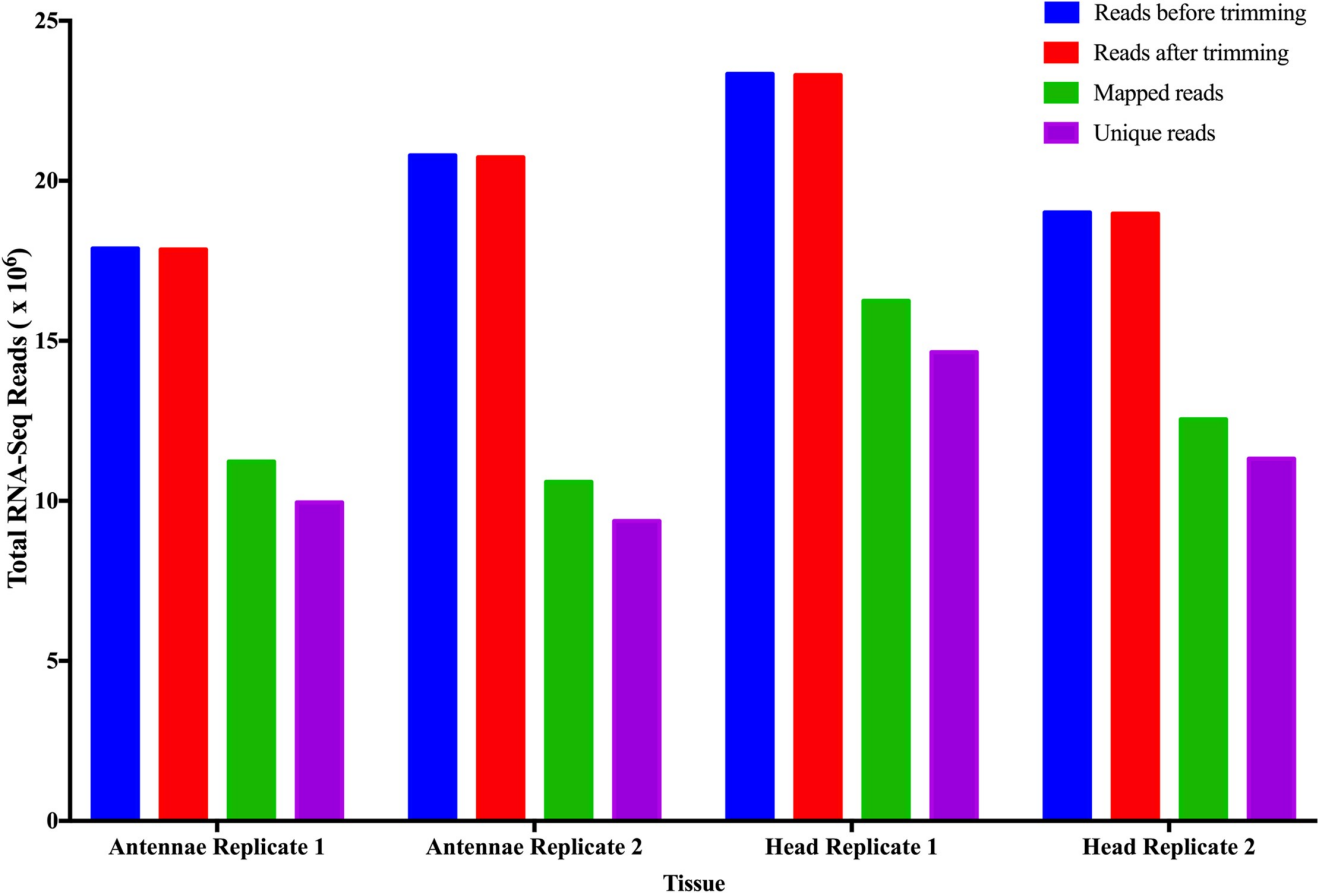
Summary of processing and mapping statistics of RNA-Seq reads from male *G*. *m*. *morsitans* antennae and head transcriptomes.

**Fig 2:**
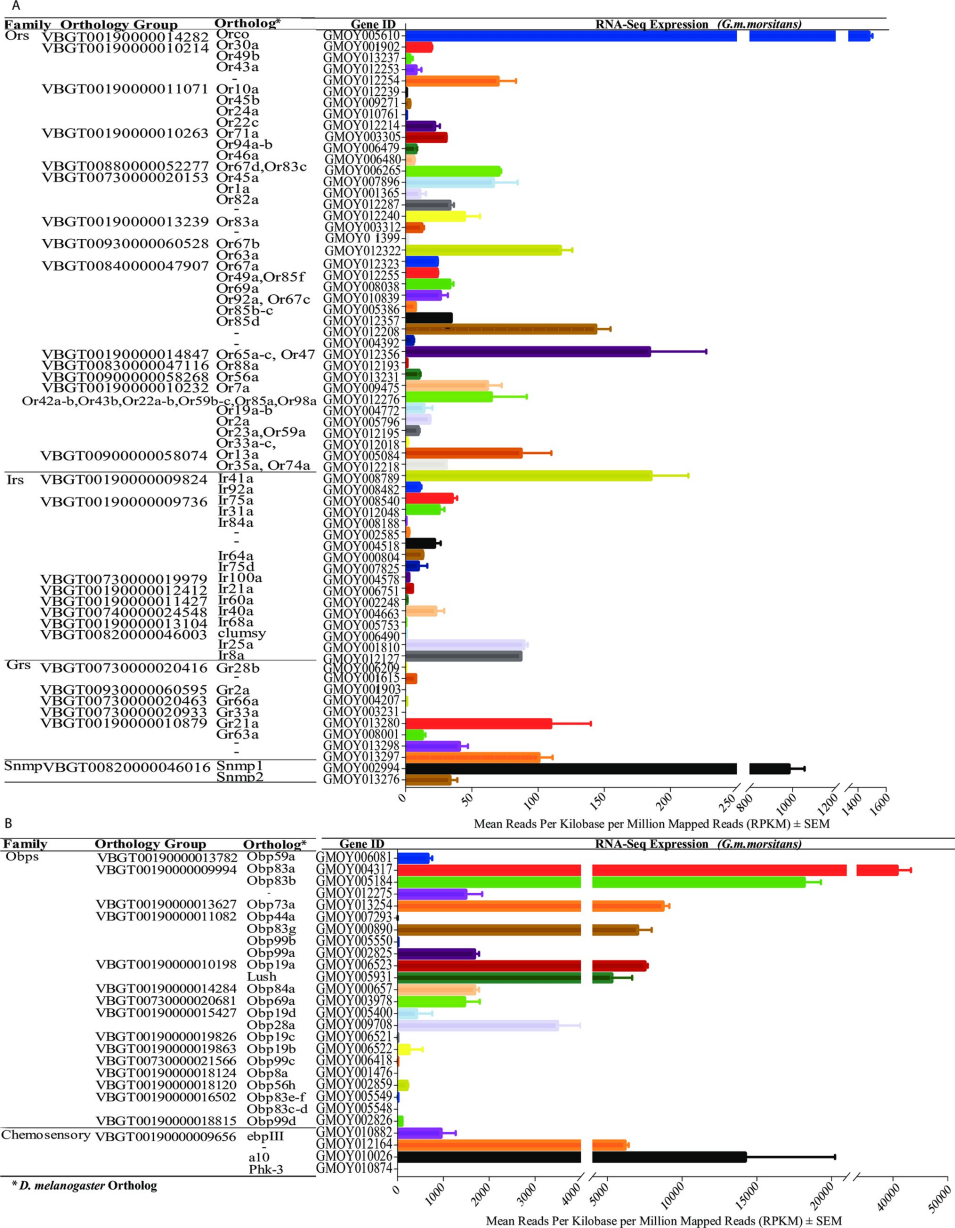
Expression profiles of *D*. *melanogaster* chemosensory gene orthologs in male *G*. *m*. *morsitans* antennae 72 hrs post feeding.

The RNA-Seq of the antennae and head libraries yielded 23.3 to 17.9 million reads from respective libraries. We successfully mapped 51.0 to 69.6% of these reads onto *G*. *m*. *morsitans* transcripts where we established about 88.4% unique mappings of the reads to specific transcripts (Fig 1). We have summarized expressions profiles of the chemosensory orthologs in Fig 2. Orco, Or56a, 65a-c, Or47b and Or67b, and three *G*. *m*. *morsitans* specific orthologs (GMOY012254, GMOY009475, and GMOY006265) were among most abundantly expressed transcripts with Or33a-c orthologs exhibiting the least expression. Expressions of the members of the significantly expanded Ors gene families were marginal. Only six Gr orthologs were expressed among which Gr21a and Gr63a orthologs (carbon dioxide receptors) [65] and related two *G*. *m*. *morsitans* specific (GMOY013297 and GMOY013298) orthologs were abundantly expressed. The putative conserved core-receptors (Ir8a and Ir25a) and Ir41a were among the most abundantly expressed Irs orthologs. All but Ir75a-c expanded Ir orthologs were expressed. Most abundantly expressed Obp orthologs include Obp19a, lush, Obp28a, Obp83a-b Obp44a and two *G*. *m*. *morsitans* specific (GMOY012275 and GMOY013254) orthologs. Among these, Obp83a-b were among the significantly expanded Obp families. Both Snmps (Snmp 1 and Snmp 2) and Csp2 were also abundantly expressed.

### Enriched pathways between male *G*. *m*. *morsitans* antennae and head libraries

Our Gene Set Enrichment Analysis (GSEA) of transcripts between the antennae and head libraries revealed several enriched pathways and processes between these tissues (Table 1, S2 Table). Our GoSlim GO analysis component of the GSEA assigned 85.4% of our transcripts to biological process, cellular components and molecular function ontologies (S2 Table). The most predominantly enriched biological processes between the antennae and head include metabolic processes, biological regulations, multicellular organismal processes, developmental processes and responses to stimuli. Most of these biological processes appeared to be localized in the membrane, macromolecular complex and nucleus cellular components, and were predominantly involved in protein binding, nucleic acid binding, ion binding and hydrolase activity molecular functions (S2 Table). More specifically, most enriched biological processes in the head were associated with vision, muscle activity and associated structural proteins and neuropeptide regulations, amino acid/nucleotide metabolism and circulatory system processes. The enriched cellular components were predominantly associated with vision and muscular functions. On the other hand, most enriched antennal biological processes were cilium-associated mechanoreceptors, chemo-sensation, neuronal controlled growth and differentiation, and regeneration/responses to stress, while enriched cellular components were associated with chemo-sensation, mechano-reception and muscular activities. Most enriched molecular functions in the head and antennae were associated with vision/muscular activities and chemo-sensation, respectively. The KEGG pathway analysis revealed enrichment of vision-associated pathways. Similarly, PANTHER pathway analysis also identified vision, in addition to neuropeptide signaling and muscular associated activities among the most enriched pathways in the head. We identified similar outcomes from our protein-protein interactions BIOGRID analysis in the head library. The Reactome pathway analysis identified vision and amino acids and derivative metabolism pathways predominating in the head transcriptome. We did not identify pathways or networks significantly enriched in the antennae library.

**Table 1:** Summary of Canonical Gene-set Enrichment Analysis (GSEA) of differentially expressed transcripts between male *G*. *m*. *morsitans* tsetse fly antennae and head transcriptomes.

Functional Database		Tissue	Annotation			Statistics					
Name	Class	Tissue	Process ID	Description	General Function	Size	L	ES	NES	P Value	FDR
Gene	Biological	Head	GO:0050953	Sensory perception of light stimulus	Vision	59	22	0.898	1.883	0.000	0.000
Ontology	Process		GO:0007186	G-protein coupled receptor signaling pathway	Vision	162	56	0.796	1.869	0.000	0.000
			GO:0032101	Regulation of response to external stimulus	Vision	101	8	0.801	1.833	0.000	0.000
			GO:0010927	Cellular component assembly involved in morphogenesis	Muscle activity	108	18	0.773	1.765	0.000	0.001
			GO:0042440	Pigment metabolic process	Vision	115	18	0.735	1.692	0.000	0.004
			GO:0009628	Response to abiotic stimulus	Vision	360	36	0.682	1.689	0.000	0.003
			GO:0003012	Muscle system process	Muscle activity	27	12	0.879	1.676	0.000	0.005
			GO:0044057	Regulation of system process	Neuropeptide muscle regulations	48	12	0.795	1.661	0.000	0.007
			GO:0043473	Pigmentation	Vision	103	18	0.706	1.610	0.003	0.025
			GO:0006730	One-carbon metabolic process	Vision	15	5	0.910	1.607	0.000	0.024
			GO:0003013	Circulatory system process	Neuropeptide regulations	40	12	0.784	1.590	0.005	0.032
		Antennae	GO:0044782	Cilium organization	Mechanoreception	62	22	-0.839	2.170	0.000	0.000
			GO:0031503	Protein complex localization	Mechanoreception	30	12	-0.849	1.903	0.000	0.001
			GO:0007606	Sensory perception of chemical stimulus	Chemo-sensation	124	57	-0.628	1.806	0.000	0.006
			GO:0035218	Leg disc development	Growth/differentiation	87	15	-0.665	1.781	0.000	0.007
			GO:0030705	Cytoskeleton-dependent intracellular transport	Mechanoreception	66	11	-0.676	1.751	0.000	0.012
			GO:0030031	Cell projection assembly	Mechanoreception	112	35	-0.624	1.742	0.005	0.011
			GO:0031099	Regeneration	Repair/response to stress	18	4	-0.828	1.711	0.000	0.015
	Cellular	Head	GO:0019898	Extrinsic component of membrane	Vision	72	11	0.870	1.891	0.000	0.000
	Component		GO:0016028	rhabdomere	Vision	34	17	0.955	1.886	0.000	0.000
			GO:0043292	Contractile fiber	Muscle activity	50	20	0.871	1.822	0.000	0.000
			GO:0015629	Actin cytoskeleton	Vision/Muscle activity	99	18	0.794	1.807	0.000	0.000
			GO:0098796	Membrane protein complex	Vision	233	10	0.690	1.689	0.000	0.001
			GO:0098858	Actin-based cell projection	Vision	22	4	0.861	1.600	0.002	0.012
			GO:0031984	Organelle sub-compartment	Vision	86	9	0.684	1.515	0.007	0.046
		Antennae	GO:0005929	Cilium	Chemo-sensation/ Mechanoreception	80	30	-0.846	2.256	0.000	0.000
			GO:0031252	Cell leading edge	Chemo-sensation	52	28	-0.811	2.005	0.000	0.000
			GO:0005815	Microtubule organizing center	Mechanoreception/Muscle activity	111	20	-0.666	1.849	0.000	0.001
	Molecular	Head	GO:0005516	Calmodulin binding	Vision/Muscle activity	43	6	0.834	1.706	0.000	0.009
	Function	Antennae	GO:0005549	Odorant binding	Chemo-sensation	49	35	-0.843	2.170	0.000	0.000
Pathway	KEGG	Head	dme04745	Phototransduction—fly—Drosophila melanogaster (fruit fly)	Vision	25	14	0.954	1.772	0.000	0.000
Analysis	Panther	Head	P00057	Wnt signaling pathway	Vision	62	8	0.827	1.749	0.000	0.000
			P00031	Inflammation mediated by chemokine and cytokine signaling pathway	Vision/Muscle activity	26	5	0.895	1.716	0.000	0.002
			P00044	Nicotinic acetylcholine receptor signaling pathway	Vision/Muscle activity	38	9	0.845	1.705	0.000	0.002
			P00016	Cytoskeletal regulation by Rho GTPase	Vision/Muscle activity	21	4	0.911	1.680	0.000	0.004
			P00004	Alzheimer disease-presenilin pathway	Vision/Muscle activity	25	5	0.848	1.593	0.005	0.026
			P00012	Cadherin signaling pathway	Muscle activity	25	3	0.839	1.585	0.003	0.025
			P00042	Muscarinic acetylcholine receptor 1 and 3 signaling pathway	Vision/Neuropeptide regulations	20	7	0.836	1.568	0.011	0.033
			P04374	5HT2 type receptor mediated signaling pathway	Vision	18	7	0.841	1.567	0.014	0.030
			P00028	Heterotrimeric G-protein signaling pathway-rod outer segment phototransduction	Vision	5	3	0.991	1.563	0.000	0.031
	Reactome	Head	R-DME-1852241	Organelle biogenesis and maintenance	Vision	38	4	0.904	1.778	0.000	0.000
			R-DME-2514856	The phototransduction cascade	Vision	12	6	0.961	1.687	0.000	0.025
			R-DME-5620920	Cargo trafficking to the periciliary membrane	Vision	15	4	0.956	1.683	0.000	0.018
			R-DME-5617833	Cilium Assembly	Vision	15	4	0.956	1.674	0.000	0.018
			R-DME-5620916	VxPx cargo-targeting to cilium	Vision	12	4	0.965	1.655	0.000	0.026
			R-DME-2514859	Inactivation, recovery and regulation of the phototransduction cascade	Vision	12	6	0.961	1.644	0.000	0.029
			R-DME-2187338	Visual phototransduction	Vision	14	6	0.957	1.644	0.000	0.025
			R-DME-76002	Platelet activation, signaling and aggregation	Vision/Muscle activity	47	9	0.784	1.640	0.000	0.024
			R-DME-71291	Metabolism of amino acids and derivatives	Metabolism	57	19	0.761	1.634	0.000	0.027
			R-DME-2672351	Stimuli-sensing channels	Vision	9	3	0.954	1.622	0.000	0.034
			R-DME-500792	GPCR ligand binding	Vision	14	4	0.920	1.618	0.002	0.034
Network Analysis	PPI_BIOGRID	Head	PPI_BIOGRID M119		Muscle activity	33	16	0.872	1.711	0.000	0.004
			PPI_BIOGRID M37		Muscle activity	71	20	0.767	1.683	0.000	0.004
			PPI_BIOGRID M80		Vision	12	8	0.957	1.643	0.000	0.017

*Non-Redundant

### Differentially expressed transcripts between male *G*. *m*. *morsitans* antennae and head libraries

**Fig 3:**
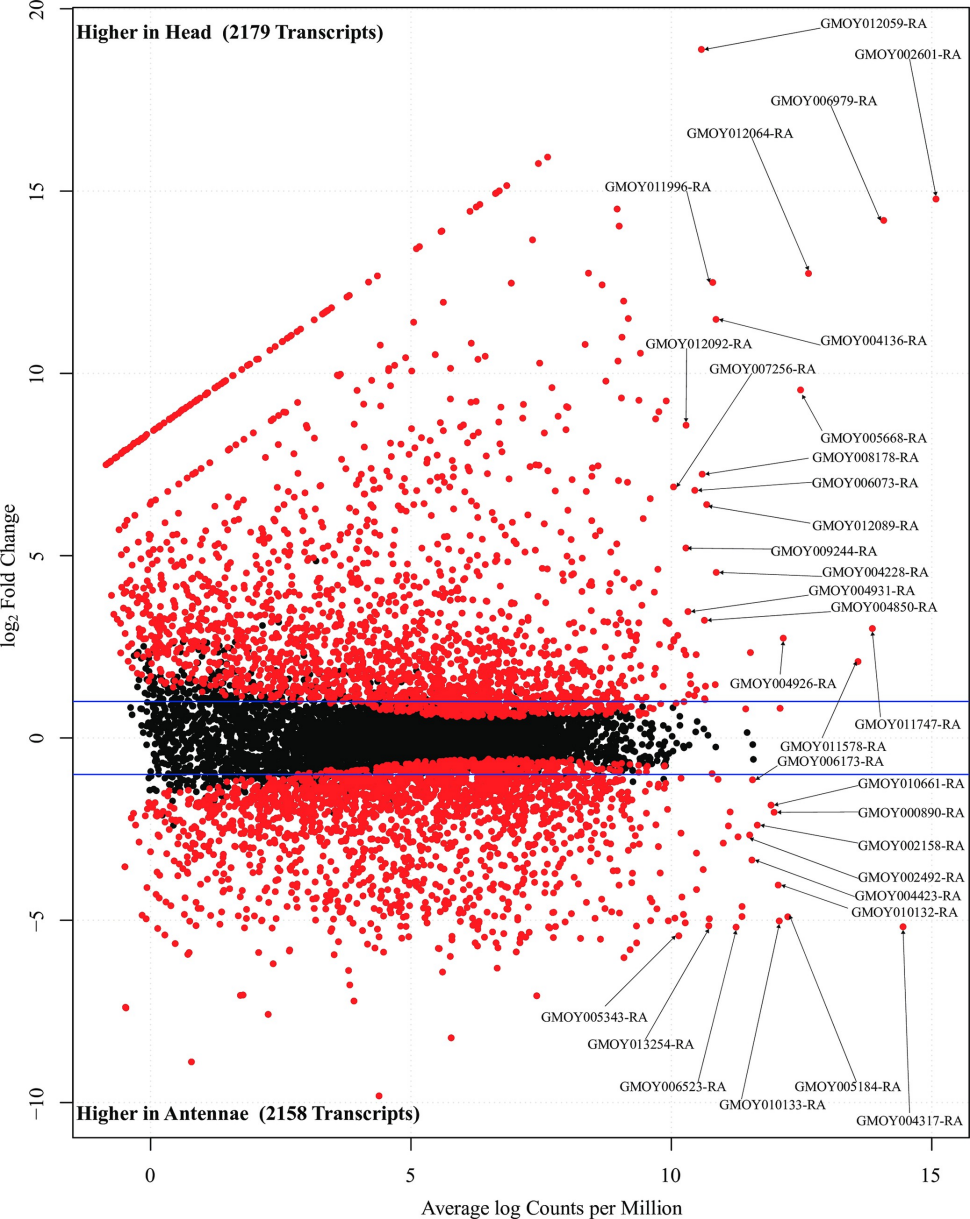
MA plot showing abundantly and differentially expressed transcripts between the male *G*. *m*. *morsitans* head and antennae transcriptomes. Dots indicate points-of-interest that display individual transcript abundance (x axis) and fold-change (y axis). Red dots indicate transcripts with fold-changes of two or more (log_2_ ≥ 1) and False Detection Rate (FDR) corrected p values of less than 0.05 (significant) between the head and antennae transcriptomes. Black dots indicate transcripts with non-significant changes between the transcriptomes.

**Fig 4:**
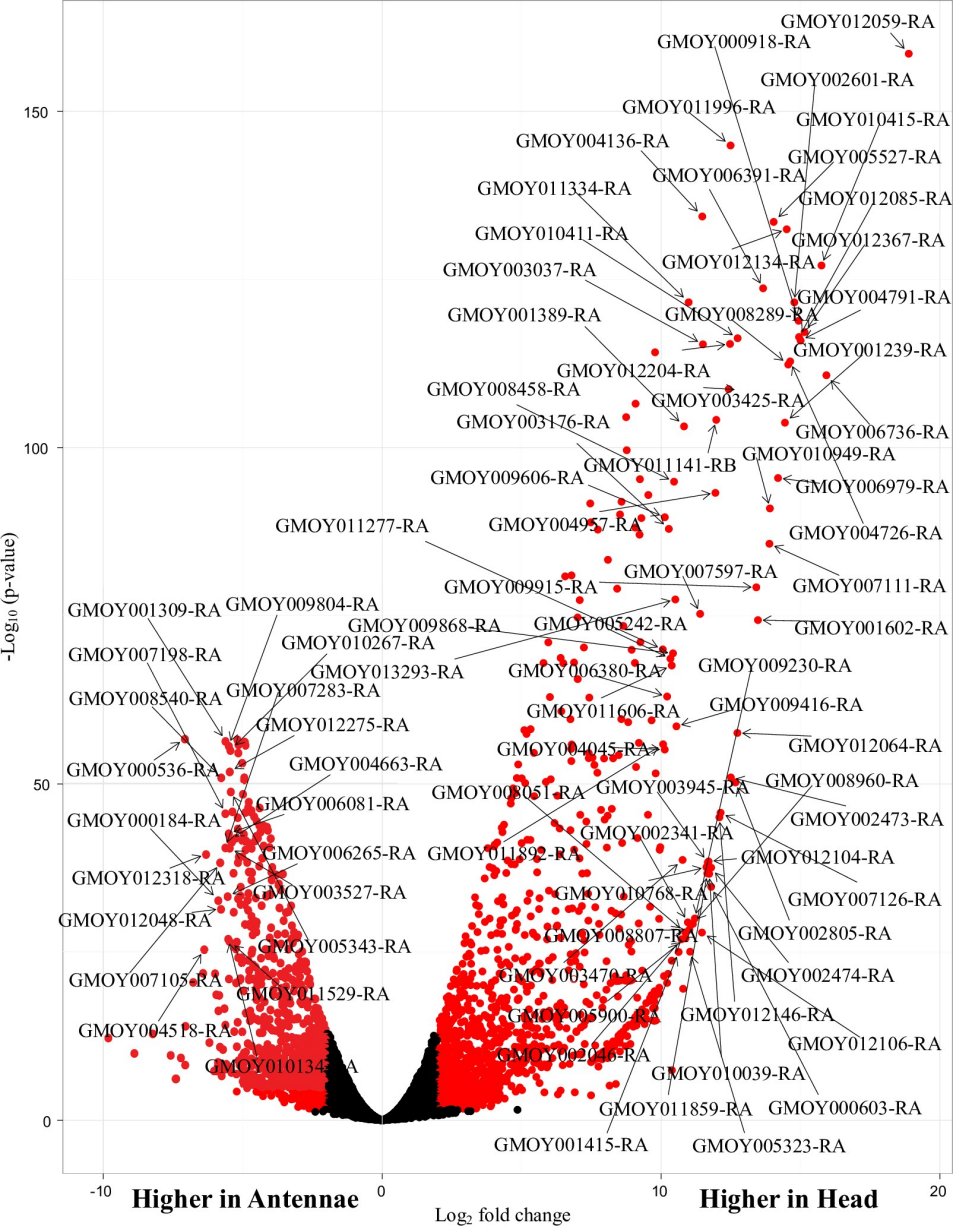
Volcano plot showing abundantly and significantly expressed transcripts between the male *G*. *m*. *morsitans* head and antennae transcriptomes. Dots indicate points-of-interest that display fold-changes (x axis) and statistical significance (-log10 of p value, y axis) in transcripts between the head and antennae transcriptomes. Red dots indicate transcripts with fold-changes of two or more (log_2_ ≥ 1) and False Detection Rate (FDR) corrected p values of less than 0.05 and are indicate transcripts with significant changes between the transcriptomes. Black dots represent transcripts with non-significant changes between the transcriptomes.

Our search for both differentially (FC > 2) and abundantly expressed (CPM > 1) transcripts between the head and antennae libraries identified 2179 and 2158 transcripts respectively differentially expressed (FDR corrected p value < 0.05) between each library as summarized in our MA plot (Fig 3). Among these transcripts, at least 52 transcripts were most differentially and abundantly expressed (log FC > 2 and Average log CPM > 10) in both libraries. These transcripts were predominantly associated with vision, iron transport, metabolism and signal transduction in the head. In the antennae, the transcripts were involved in odor sensing and clearing, fatty acid synthesis and regulation of feeding behavior and locomotor activity (S3 Table). Analysis of both differentially (FC) and significantly expressed (p-value) transcripts between the head and antennae libraries identified 49 and 61 transcripts as most significantly expressed (FC >10 or <-5, and–log_10_ p-value > 25) in the head and antennae libraries respectively as summarized in our volcano plot (Fig 4). Overall, about 40 and 52 percent of the transcripts were associated with vision (head) and chemo-sensation (antennae) respectively. Most significantly expressed transcripts in the head library were functionally associated with energy mobilization, feeding, immunity, cytoskeleton integrity, amino acid metabolism, endocrine signaling and neuronal development and support. In the antennae, most significantly expressed transcripts were functionally associated chemo-sensation, metabolism, and cell proliferation, regulation of gene expression, signal transduction, anatomical integrity, neuron integrity/development and mechanoreception (S3 Table).

### Differential expression of chemosensory gene transcripts between male *G*. *m*. *morsitans* antennae and head libraries

**Fig 5:**
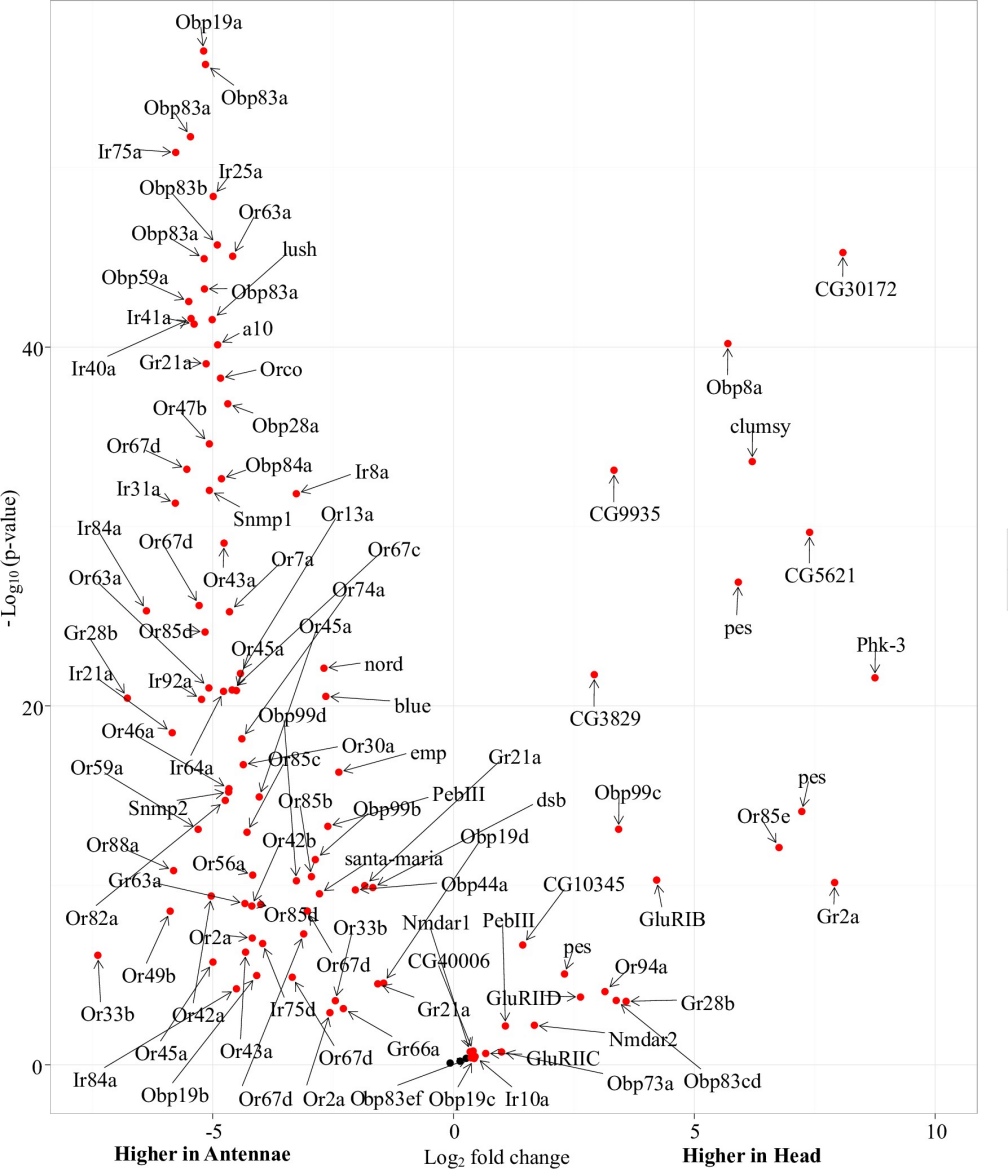
Volcano plot showing abundantly and significantly expressed chemosensory gene orthologs between the male *G*. *m*. *morsitans* head and antennae transcriptomes. Dots indicate points-of-interest that display fold-changes (x axis) and statistical significance (-log10 of p value, y axis) in transcripts between the head and antennae transcriptomes. Red dots indicate transcripts with fold-changes of two or more (log_2_ ≥ 1) and False Detection Rate (FDR) corrected p values of less than 0.05 and are indicate transcripts with significant changes between the transcriptomes. Black dots represent transcripts with non-significant changes between the transcriptomes.

When we considered fold change greater than 1.25 as of biological chemosensory significance [64], most (> 90%) chemosensory transcripts showed significantly higher expressions in the antennae than in the head (Fig 5). Among these, significantly expressed chemosensory transcripts (p-value < 1e-20) in the antennae include several Obp (Lush, Obp19a, Obp28a, Obp59a, Obp83a/b and Obp84a), Ir (Ir25a, Ir31a, Ir40a, Ir41a, Ir64a, Ir75a, Ir76b, Ir84a, Ir8a and Ir92a), Or (Orco, Or7a, Or13a, Or43a, Or45a, Or47b, Or63a/c/d and Or85d), Gr (Gr21a), Csp [Csp2 (a10) and Csp4 (Phk-3)] and Snmp1 orthologs. Specifically, most significantly expressed transcripts were predominantly Obp orthologs. On the other hand, we identified a subset of obp (Obp8a, Clumsy, Obp99c Obp83cd), Or (Or85e, Or71a), Grs (Gr2a, Gr28b) and Csp4 (Phk-3) orthologs with significantly higher expression in the head than in the antennae libraries.

## Discussion

In this study, we profiled expansions of chemosensory gene orthologs among six tsetse fly species/subspecies (*G*. *pallidipes*, *G*. *m*. *morsitans*, *G*. *austeni*, *G*. *p*. *gambiensis*, *G*. *f*. *fuscipes* and *G*. *brevipalpis*) and employed RNA-seq to discern differential expressions of the orthologs and associated proteins in antennae and head tissues male *G*. *m*. *morsitans*. Our café analysis for gene expansion revealed significant accelerated expansion of 4-methyl phenol and 4-propyl phenol responsive Or71a [66] in *G*. *m*. *morsitans*. The 4-methyl phenol and 4-propyl phenol are known *G*. *m*. *morsitans* and *G*. *pallidipes* attractants present in natural ox odor [17,67]. These findings probably account for the observed differential responses of these species to synthetic blends of these odors [68]. On the other hand, expansions of Ir75a,d, Ir31a, Ir84a and Ir64a orthologs in *G*. *m*. *morsitans* suggest differential odor-tuning and responses to acetic acid and 2-oxopentanoic acid in this species [69–73]. Acetic acid component of the vertebrate breath is an attractant of most hematophagous vectors while 2‐oxopentanoic acid elicit a landing response from *Anopheles gambiae* [74]. Whether there is enhanced attraction and landing behavior in *G*. *m*. *morsitans* in the presence of these kairomones remains to be determined. Expansion of Ir84a in *G*. *m*. *morsitans* may also indicate enhanced response to phenylacetaldehyde and male-male courtship [75] in this tsetse fly specie relative to the other species. Expansion of hunger responsive Obp83a ortholog [76] in *G*. *m*. *morsitans* suggest enhanced host seeking persistence in this specie relative to the other species. The *G*. *pallidipes* appears to be characterized by potentially muted responses to feeding stimulating hydroxycinnamic acids linked to missing Or71a [77], but enhanced responses to butanol, 2-heptanone and ketones lactones and phenolic compounds associated with the expanded Or49a [78,79], Or67a [80], Or85f [81] and Or85c [82] orthologs. The responses to butanol, lactones, ketones and phenolic compounds have been evaluated in development of baits used routinely in field control of *G*. *pallidipes*. Carbon dioxide receptors Gr21a and Gr63a orthologs [65] were expanded in *G*. *f*. *fuscipes* and most abundantly expressed in male *G*. *m*. *morsitans* antennae. These findings are indicative of the heavier investment by *G*. *f*. *fuscipes* than other tsetse flies in carbon dioxide detection and consequently host location [83]. The potential impact of the expansion (in *G*. *f*. *fuscipes*) of the Ir21a required for cool avoidance behavior [84] is not clear, but may be tied to the humid and warm habitat preference in the *G*. *f*. *fuscipes* lacustrine habitats. The Gr64a-f, Gr5a and Gr43a sugar receptor orthologs [85,86] were conspicuously absent in tsetse flies, consistent with our previous finding [40], a phenomenon attributable to exclusive sugar deficient blood diet in tsetse flies. The *G*. *brevipaplis* specific expansions of hygrosensory behavior mediating Ir68a ortholog [87] suggest potential behavioral responses to these and related odor cues specific to this tsetse fly. We did not identify expansion of Or67d in tsetse flies, contrary to previous reports [39,40].

We identified several missing/absent or conserved tsetse fly species specific orthologs with potential implications on respective tsetse species phenotypes. Absent Gr33a ortholog responsive to nonvolatile repulsive chemicals, including N,N-diethyl-meta-toluamide (DEET) [88,89] in *G*. *brevipalpis* and marginal expression of Gr66a ortholog in male *G*. *m*. *morsitans* antennae, suggest diminished responses in these species to some repellents. This phenomenon is further supported by absence of another caffeine and DEET responsive Gr32a ortholog [88,89] and courtship pheromone associated Gr68a ortholog [90] in *G*. *brevipalpis* and *G*. *m*. *morsitans*. The missing Ir76b ortholog in four tsetse fly species (*G*. *p*. *gambiensis*, *G*. *m*. *morsitans*, *G*. *pallidipes* and *G*. *brevipalpis*) suggests that these tsetse species may have reduced responses to Ir76b ortholog mediated feeding preferences for amino acids [73] relative to remaining tsetse fly species. The conspicuous absence of Obp56a,d,e orthologs in tsetse flies, point to possible reduction in their responses to the associated pheromones [91]. Geosmin responsive Or56a ortholog [92] was most abundantly expressed Or after Orco in the *G*. *m*. *morsitans* antennae. Since Geosmin is a microbial odorant that alerts flies of presence of harmful microbes and induces avoidance behavior [92], the findings suggest potential repellence of tsetse flies by Geosmin and associated compounds, which can form a basis for a search for tsetse fly specific repellents. Conserved Gr2a, Gr28b and Gr66a orthologs across most species supports a notion of general aversion of salts [93], caffeine, DEET and some amino acids (theophylline, threonine and valine) [88,94–97] among the vectors. The Snmp1 ortholog associated with detection of pheromones appears to be conserved across all the tsetse fly species, which in concert with similarly conserved Or67d and Orco orthologs, are functionally associated with detection of lipid-derived pheromones [98,99]. Other conserved pheromone responsive orthologs, include male-specific pheromone 11-cis-vaccenyl acetate (cVA) responsive lush and Obp19a [100] (absent in *G*. *austeni*) and l-carvone, 2-heptanone and acetophenone responsive Obp83a [101]. Lush, Or67d, Or83c and Obp83a were predominantly expressed in male *G*. *m*. *morsitans* antennae. We identified Ir93a ortholog in *G*. *austeni* contrary to previous findings [40]. Overall, we identified potential tsetse fly specific receptors and semiochemicals/ligands for downstream functional validations that can be employed to expand the toolbox of tsetse fly attractants, repellents and regulators.

Our gene and pathway enrichment analyses suggest that male *G*. *m*. *morsitans* head and antennae are predominately involved with vision and olfaction (odor sensing and clearing) respectively. In addition to the classical and canonical olfaction pathways, we also established fatty acid synthesis and associated xenobiotic responsive cytochrome P450 (Cyp6g1/2, Cyp304a1) and Glutathione S transferase pathways preferentially enriched in the antennae. Similar observations have been made in cutworm moth (*Agrotis ipsilon*) antennae [102] and may indicate significant investment in odor/pheromone clearing [103], probably as a strategy for faster desensitization of antennae responses in the absence or disengagement with relevant cues. Other enriched pathways and transcripts included lush, lush-like Obp19a, Obp28a and Obp83a/b, Obp84a, Or7a and Snmp1 that are associated with responses to pheromones [91,104]. The antennae transcriptome appears to be dominated with abundant, differentially expressed Ir75a-c, Ir31a, Ir84a, Ir41a, Ir92a and Gr21a orthologs, functionally associated with responses to various odor cues including acetic acid, 2-oxopentanoic acid [70–72], pyridine, 1,4-diaminobutane, cadaverine, spermidine, pyrrolidine [72], phenylacetyaldehyde [26], ammonia [20] and carbon dioxide [65]. Some of the cues, such as butanol, carbon dioxide and acetic acid are documented odor cues in the breath of the tsetse fly vertebrate hosts and are actively employed by tsetse fly in host location [10,15], suggesting that the rest might perform similar functions in nature.

The antennae were also enriched with transcripts associated with cilium mechanoreceptors/locomotor activity, indicating possible significant role of antennae in the detection of kinetic energy (energy of movement, e.g. touch, sound, vibration, changing pressure) or potential energy (e.g. gravity) and hence guiding physical orientation of the fly. Stress induced neuronal controlled growth and differentiation and regeneration pathways were also enriched in the antennae, suggesting important role of the antennae in modulating responses of the fly to fluctuations in oxygen levels, temperature and redox state [105]. In addition to vision gene, the head was enriched with muscle and associated structural proteins, and energy mobilization potentially associated with feeding, as well as neuropeptide regulations associated with modification of nervous and endocrine systems. Most differential and abundantly expressed head specific chemosensory transcripts were also functionally associated with feeding. These included Obp8a involved in food perception [106] and host location [107], and Gr28a/b and Gr2a linked to regulation of aversion to high-salt associated diet [93]. Phenotypic roles of other head-specific chemosensory transcripts, such as Csp2 (a10) and Csp4 (Phk-3), Clumsy, Obp99c Obp83cd, Or85e, Or71a and Csp4 (Phk-3), remain to be elucidated. Other than vision, olfaction and associated molecular processes, other processes appear to dominate physiological and molecular functions in the head and antennae libraries, respectively, indicating other functional roles of these tissues. Since these tissues (antennae and head) where extracted in the morning, the transcriptional responses coincided with the peak activity of the tsetse flies and hence reflect chemosensory and visual processes associated with host finding behavior predominant in that duration. Since our gene analyses were focused on antennae from male *G*. *m*. *morsitans*, our gene expression results were potentially biased toward male tsetse flies and *G*. *m*. *morsitans* subspecies. It would therefore be prudent to further assess for similar response in the remaining five tsetse fly species/subspecies, both gender and at different physiological states that influence their olfactory responses.

## Conclusions

We identified tsetse fly specific chemosensory gene orthologs and their putative ligands, as potential candidates for downstream functional genomic and field validations. The validations could yield new tsetse fly attractants, repellents and pheromones with potential in incremental improvements of current tsetse fly control strategies. We also identified major sensory pathways and processes potentially active in the tsetse fly antennae and head that can be exploited in modulating tsetse fly behavior.

## Supporting information

S1 Fig Principal Component Analysis (PCA)-based clustering of gene orthologs showing differences in number of expanded/contracted orthologs between the six tsetse fly species. (A) Clustering of chemosensory orthologs between tsetse species (B) Clustering of individual orthologs within chemosensory gene families.
(TIF)

S1 Table Counts of chemosensory gene orthologs among fruit fly (*D*. *melanogaster*) and selected tsetse fly species. (XLSX)

S2 Table Canonical Gene-set Enrichment Analysis (GSEA) Gene Ontology, Kyoto Encyclopedia of Genes and Genomes (KEGG), Panther and Reactome pathways, and Protein-Protein Interactions BIOGRID network statistics for the differentially expressed transcripts between male *G*. *m*. *morsitans* antennae and head transcriptomes. (XLSX)

S3 Table Annotations of most abundantly or significantly differentially expressed transcripts between male *G*. *m*. *morsitans* antennae and head transcriptomes. (XLSX)
