# Supplementary figures and images for: Expansions of chemosensory gene orthologs among selected tsetse fly species and their expressions in Glossina morsitans morsitans tsetse fly
Source: PLoS Negl Trop Dis. 2020 Jun 26;14(6):e0008341. doi: 10.1371/journal.pntd.0008341 (PMC7347240; doi:10.1371/journal.pntd.0008341)

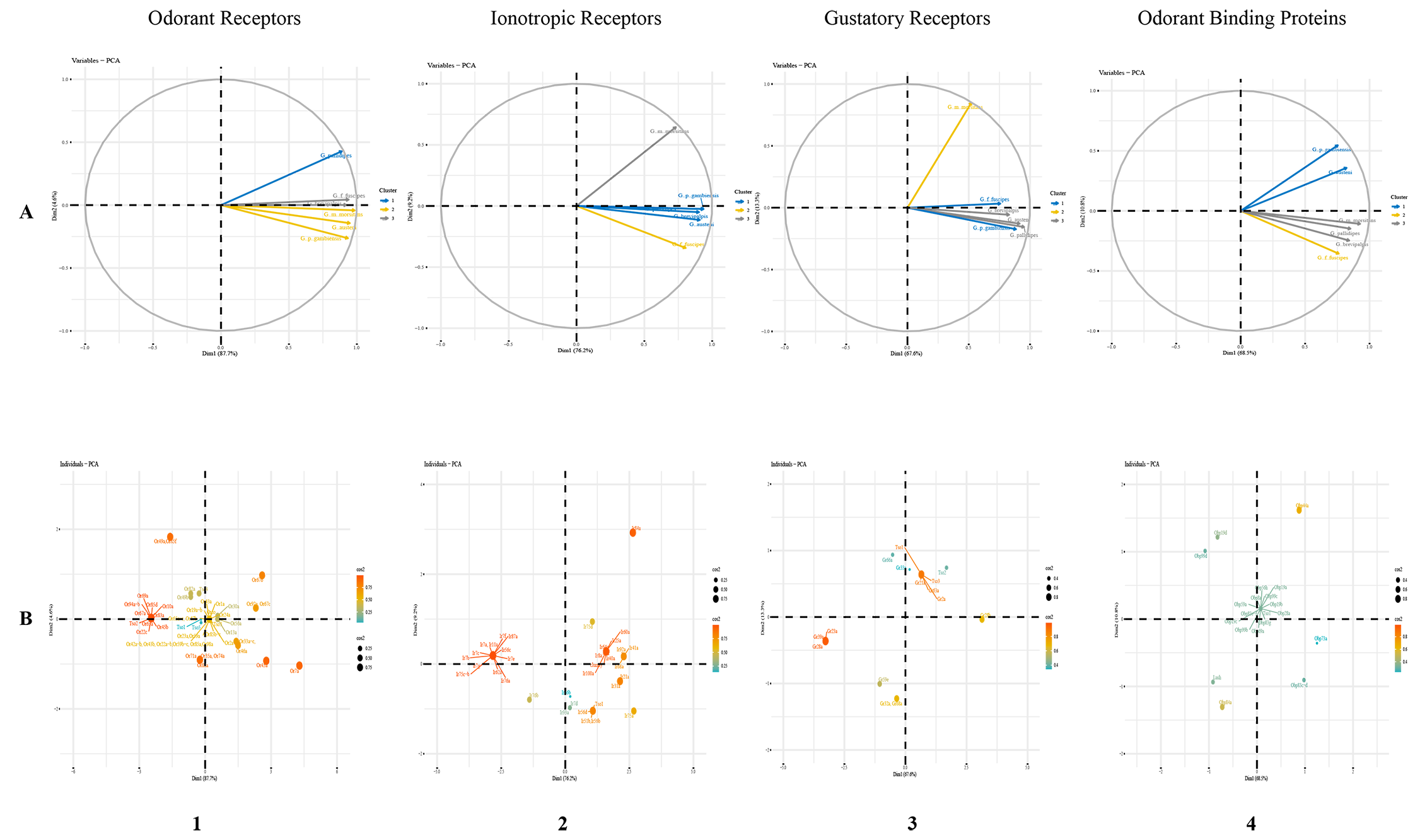

Supplement: S1 Fig — (A) Clustering of chemosensory orthologs between tsetse species (B) Clustering of individual orthologs within chemosensory gene families. (TIF) [file pntd.0008341.s001.tif]
